# Short-course R-CHOP followed by ^90^Y-Ibritumomab tiuxetan in previously untreated high-risk elderly diffuse large B-cell lymphoma patients: 7-year long-term results

**DOI:** 10.1038/bcj.2016.29

**Published:** 2016-05-13

**Authors:** V Stefoni, B Casadei, C Bottelli, G Gaidano, C Ciochetto, M G Cabras, M Ansuinelli, L Argnani, A Broccoli, L Gandolfi, C Pellegrini, P L Zinzani

**Affiliations:** 1 University of Bologna, Institute of Hematology ‘L. e A. Seràgnoli', University of Bologna, Bologna, Italy; 2 Division of Hematology, Presidio Ospedaliero ‘Ospedali Civili', Brescia, Italy; 3 Division of Hematology, Amedeo Avogadro University of Eastern Piedmont, Novara, Italy; 4 Division of Hematology, Azienda Ospedaliera Città della Salute e della Scienza di Torino, Torino, Italy; 5 Division of Hematology, Cagliari Hospital, Cagliari, Italy; 6 Department of Cellular Biotechnologies and Hematology, ‘La Sapienza' University, Roma, Italy

## Abstract

An update at 7 years was conceived for our multicenter phase II study in which 55 elderly high-risk untreated diffuse large B-cell lymphoma patients were treated with ^90^Y-ibritumomab tiuxetan after a short course of rituximab, cyclophosphamide, doxorubicin, vincristine and prednisolone (R-CHOP) as long-term follow-up analyses of this combined therapeutic modality are lacking. The overall response rate to the entire regimen was 80%, including 73% (40/55) of complete response (CR) rate and 7% (4/55) of partial response rate. At the time of writing, 24/55 (43.6%) patients experienced a progression disease and 20 of 40 (50%) patients who obtained a CR are still alive in continuous CR. With a median follow-up of 7 years, the disease-free survival was 43.3% and the progression-free survival was 36.1%. The overall survival at 7.9 years was 38.9% (27 deaths mainly because of lymphoma). Two patients developed secondary hematological malignancies, an acute myeloid leukemia and a myelodysplastic syndrome, at 4 and 3 years from radioimmunotherapy, respectively. Our data confirm the feasibility, efficacy and safety of four cycles of R-CHOP followed by radioimmunotherapy consolidation even in the long term: this combination allows dispensing less chemotherapy in a frail group of patients without invalidating response quality and duration.

## Introduction

The radiosensitivity of lymphomas, such as in particular the local, specific targeted delivery of radiation radioimmunotherapy (RIT), is an interesting therapeutic option for lymphoma patients. RIT represents an attractive alternative approach for several reasons. First, lymphoma is known to be radiosensitive; second, radiolabeled antibodies can kill target lymphoma cells via both immunological lysis and release of β-rays from isotopes attached to the antibodies; third, unconjugated antibodies, such as rituximab, cannot kill lymphoma cells if the patient's immune system is abnormal, decreased expression of the target antigen is present or the tumor is difficult to approach, whereas RIT can kill tumor cells via a cross fire or bystander effect.

Encouraging results of RIT in low-grade lymphomas also led to its investigation as a possible therapy in aggressive non-Hodgkin's lymphoma. Although high-dose chemotherapy followed by peripheral stem cell transplantation is a recommended standard in cases with a partial response or relapsing diffuse large B-cell lymphoma (DLBCL), many patients are not eligible for this procedure because of advanced age or comorbidities. Thus, RIT may be an interesting alternative therapeutic option for this subset of patients.

RIT, in particular the ^90^Y-ibritumomab tiuxetan (^90^Y-IT), has demonstrated efficacy in relapsed/refractory DLBCL, with promising response rates and durable response.^1, 2, 3^ In the initial phase I–II trials, responses were seen in 43% of the 14 patients with DLBCL, including complete remission (CR) in 29%.^1^ Gordon *et al.*^4^ provided additional data supporting the safety and the efficacy of ^90^Y-IT in patients with DLBCL: for 12 patients who had a median of two prior regimens and who were enrolled in this phase I–II trial, the response rate was 58%, including 33% CRs. A multicenter phase II study has assessed the role of ^90^Y-IT in 104 elderly patients with relapsed or refractory DLBCL who were not eligible for high-dose treatment, mainly because of advanced age.^3^ The overall response rate was 44% and was noted to be higher in patients who did not have prior therapy with rituximab compared with those who had been previously treated in first line with rituximab and chemotherapy.

In addition, data from phase II studies conducted in DLBCL and in mantle cell lymphoma suggested that ^90^Y-IT is also effective as consolidation treatment after chemotherapy or chemoimmunotherapy.^5, 6, 7, 8^ One of these represented our experience: a phase II study in 55 high-risk elderly patients treated in first line with an abbreviated (four cycles than six) course of rituximab, cyclophosphamide, doxorubicin, vincristine and prednisolone (R-CHOP) followed by ^90^Y-IT consolidation.^8^

Long-term evaluations of both efficacy and safety are now needed to best integrate and place this option into current elderly DLBCL patient treatment algorithms. The first report we published referred to efficacy, safety and outcomes at 18 months:^8^ we now present the updated results of this study after a median follow-up of 7 years.

## Patients and methods

### Patients

Fifty-five elderly patients (aged >65 years or 60–65 years, but not eligible for high-dose therapy with autologous stem cell transplantation) with high-risk DLBCL were enrolled between December 2006 and October 2008 at 7 Italian institutions. The criteria for patient eligibility have been previously reported.^8^ Briefly, patients had to be previously untreated and with stage II (with bulky disease), stage III or stage IV DLBCL,^9^ expressing the CD20 antigen; they had to have a World Health Organization (WHO) performance status ⩽2 and a high intermediate or high age-adjusted International Prognostic Index score.^10^

All the patients underwent a full medical history, physical examination, complete blood tests, computed tomography (CT) scan of neck, chest, abdomen and pelvis, whole-body positron emission tomography (PET) scan and a bone marrow biopsy. Patient characteristics are summarized in Table 1. All patients were notified of the investigational nature of this study and signed a written informed consent approved in accordance with institutional guidelines, including the Declaration of Helsinki; the study was approved by each institutional review board.

### Study design

Treatment schedule was represented by the standard R-CHOP chemotherapy every 21 days for 4 cycles; standard doses of 375 mg/m^2^ rituximab, 750 mg/m^2^ cyclophosphamide, 50 mg/m^2^ doxorubicin and 1.4 mg/m^2^ (maximum total dose of 2.0 mg) vincristine were administered intravenously on day 1; 100 mg/day prednisone was given orally for 5 days for each cycle, starting from day 1. After the completion of the fourth cycle of R-CHOP regimen, patients were restaged by CT scan, PET scan and bone marrow biopsy. Patients were eligible for ^90^Y-IT if at least in partial response (PR) after induction, with normal platelet and granulocyte counts and a bone marrow infiltration ⩽25%.

After the restaging procedures, eligible patients received one course of ^90^Y-IT within 12 weeks. It consisted of an infusion of rituximab at 250 mg/m^2^ on day 1 and a subsequent infusion at the same dose on day 8; this second infusion of rituximab was then followed by a weight-based dose of ^90^Y-IT, administered at the dose of 11.1 MBq/kg (0.3 mCi/kg) for patients with pretreatment platelet counts ranging from 100 × 10^9^ to 149 × 10^9^/l, and 14.8 MBq/kg (0.4 mCi/kg) for those with counts of 150 × 10^9^/l or higher (^90^Y-IT was routinely administered on an outpatient basis).

### Follow-up procedures

Strict follow-up procedures were shared among the investigational sites at the time of the start of the study. The original protocol was amended to extend follow-up until 9 years from end of treatment. Disease status was evaluated again 3 months after RIT through physical examination, bone marrow biopsy (if still positive after induction chemoimmunotherapy), CT and PET scan; other clinically relevant information, such as the development of febrile neutropenia, the use of antibiotics or granulocyte-colony stimulating factor or blood transfusion during cytopenia and the presence of any extra-hematologic toxicity, were recorded.

Patients' follow-up assessment included: blood count and physical examination every 3–4 months for the first 2 years and then every 6 months for the following 3 years, CT scan every 6 months for the first 2 years, followed by physical examination and annual imaging studies with CT or PET/CT up to 9 years from treatment completion. The determination of tumor response was based on the revised response criteria for malignant lymphoma.^11^

### End points

Endpoints of long-term efficacy were progression-free survival, disease-free survival and overall survival. Overall survival was calculated from start of R-CHOP treatment to the date of death because of any cause and was censored at the last date the patient was known to be alive. Disease-free survival was calculated for CR patients from first documentation of response to the date of relapse or death because of lymphoma or acute toxicity of treatment, whereas progression-free survival was calculated for all patients from the start of treatment to relapse or death because of any cause.^11^ End points of late toxicity were the incidence and the time of occurrence of secondary malignancy both hematologic and nonhematologic. The crude incidence of secondary malignancies was calculated as the proportion of patients diagnosed with secondary malignancy in the entire study population. Analyses were conducted on an intention-to-treat basis.

## Results

At the time of reassessment after 4 cycles of R-CHOP-21, overall response rate was 89%, with 32 out of 55 (58%) patients achieving a CR and 17 out of 55 (31%) patients achieving a PR; the remaining 6 patients had progressive disease. Out of 55 patients (more specifically, all the patients with CR and 16 patients with PR), 48 were deemed eligible for subsequent consolidation with ^90^Y-IT. The remaining patient with PR showed a lymphoma progression 2 weeks before radioimmunotherapy, and was then considered ineligible for consolidation treatment.

**Figure 1:**
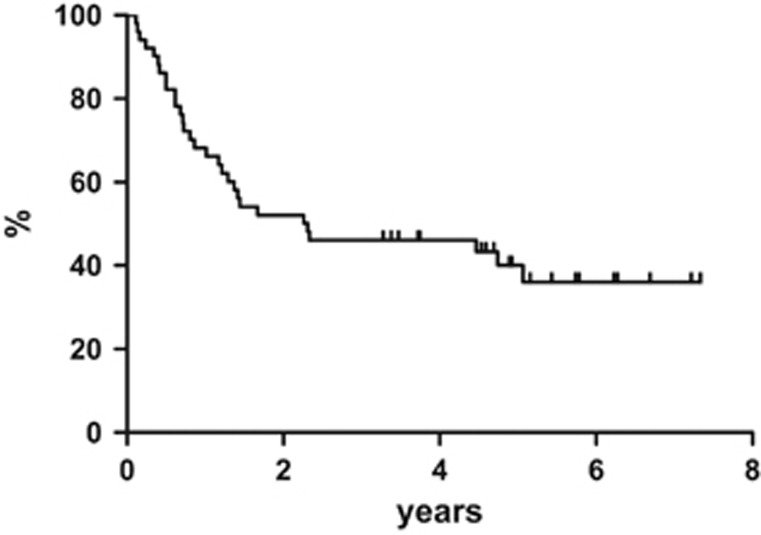
Progression-free survival.

**Figure 2:**
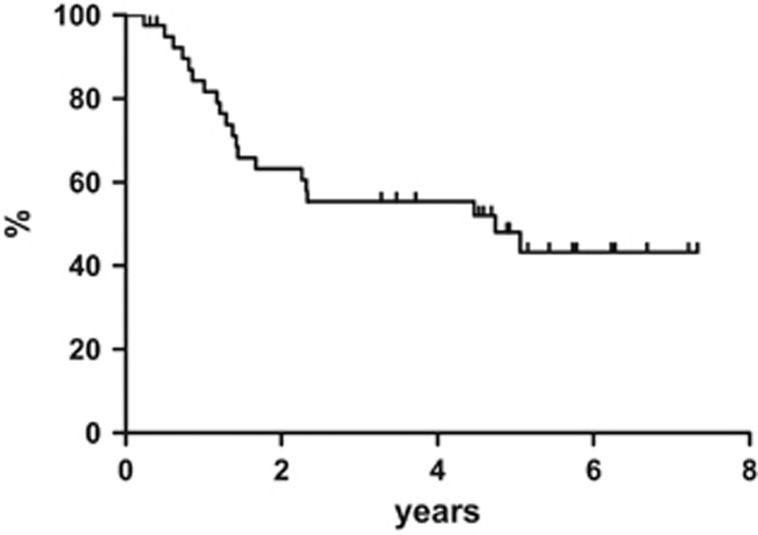
Disease-free survival.

**Figure 3:**
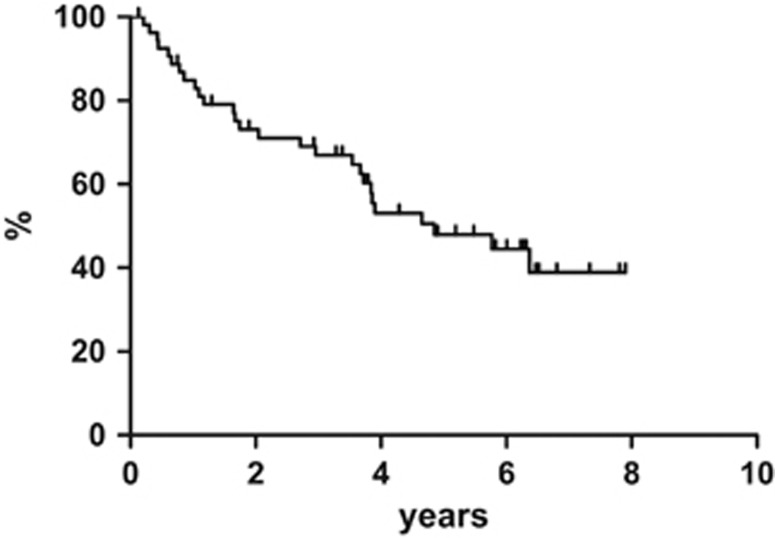
Overall survival.

At the end of the entire treatment regimen (4 cycles of R-CHOP regimen plus ^90^Y-IT), 40 out of 55 (73%) patients obtained CR and 4 subjects achieved PR. In the specific instance of when patients who were in CR right after R-CHOP maintained their disease status, 8 PR patients improved the response from partial to complete, 4 patients remained in PR and the other 4 PR had a disease progression. All patients completed the scheduled follow-up period. At a median follow-up of 84 months (range, 12–95 months), the 7-year progression-free survival was estimated to be 36.1%, with 35 events on an intention-to-treat basis (Figure 1). The 7-year disease-free survival was 43.3% (Figure 2) and the 7-year overall survival was estimated to be 38.9% (Figure 3), with 27 deaths.

Please refer to the previous publication regarding the toxicity of the regimen.^8^ Regarding the late hematological side effects, secondary malignancies occurred in 2 (3.6%) patients: one reported myelodysplastic syndrome and the other secondary acute myeloid leukemia, developed after 36 and 48 months following RIT, respectively (the patient with acute myeloid leukemia died).

Currently, 20 patients (50%) are in continuous CR after >5 years of follow-up. Twenty-four (20 CR and 4 PR) patients relapsed during the follow-up period and all died because of lymphoma progression. All the 24 relapsed patients underwent a second-line treatment and 3 of them are now in CR, whereas all the other had a brief response (<12 months) or no response to subsequent therapies. Autologous stem cell transplantation was attempted in 3 out of 24 patients: the attempt failed in two subjects, whereas the third patient is currently in CR after autologous stem cell transplantation.

## Discussion

Despite markedly superior outcomes in first-line treatment following the addition of rituximab to the CHOP regimen, with dose-dense/dose-intense regimens also displaying a potential role,^12, 13, 14, 15, 16^ the prognosis for DLBCL patients aged >60 years still remains poor. At least 30 to 50% of these patients with an advanced-stage disease will fail to attain a remission with primary therapy, or will experience disease relapse after achieving a remission.

As early results have indicated that ^90^Y-IT consolidation has a favorable tolerability profile, with low infection rates and a manageable hematologic toxicity, we carried out this study with CHOP plus rituximab followed by ^90^Y-IT in untreated elderly DLBCL patients, also reducing the number of CHOP cycles from six to four. The aim was to utilize all the therapeutic approaches—chemotherapy, immunotherapy, RIT—reducing conventional chemotherapy and probably its related toxicity, hematologic and nonhematologic, in elderly patients.

Independently by the different front-line treatment, it should be very important to have an update on the long-term follow-up of these studies because the disease course of elderly high-risk DLBCL patients is characterized by a high rate of relapse.^12, 13, 14, 15, 16^ For this reason, this report updated long-term efficacy and toxicity results of this sequential treatment and it represents, among the other published data on the role of ^90^Y-IT consolidation in DLBCL, the first long-term analysis.

With a median follow-up of 84 months, the 7-year disease-free survival was 43.3%, and thus means that 20/40 patients are still in continuous CR after >5 years in a subset of high-risk elderly DLBCL patients: it is possible to observe a real curve plateau after 5 years, suggesting that a proportion of patients may be salvaged.

Regarding late toxicity, we observed 3% of myelodysplastic syndrome/acute myeloid leukemia. The crude incidence of treatment-related myelodysplastic syndrome/acute myeloid leukemia has been reported to range from 0 to 12% with conventional dose chemotherapy or radiotherapy, and our 3% is within this range.^17^ In the long-term update of the LNH-98.5 trial comparing DLBCL treated with CHOP with R-CHOP, 10.8% of patients developed another malignancy since the enrollment.^18^ Thus, no difference can be acknowledged in the safety profile between R-CHOP alone and R-CHOP followed by RIT. Rate of mobilization failure in our population is two thirds, but it is difficult to draw any conclusion with these small numbers or to make any inference about the involvement of RIT in this issue.

Considering the long-term update of the LNH-98.5 trial comparing DLBCL treated with CHOP with R-CHOP, we achieved same long-term results.^18^ To note, a lower incidence of secondary malignancy in our sample occurred. We registered a 50% conversion rate from PR to CR after RIT with, in addition, these patients still being in CR; in the challenging group of elderly patients this feature could prevent the need of further treatments.

Although the pivotal study of R-CHOP regimen in advanced elderly DLBCL patients utilized eight cycles of chemotherapy,^14^ now many experts choose a six-cycle approach as standard treatment, relying principally on the German experience that demonstrated the equivalence of six versus eight cycles of R-CHOP given every 14 days.^12^

On the basis of the long-term update of our data, there is confirmation on the role of ^90^Y-IT in elderly high-risk DLBCL patients. In addition, this sequential treatment is characterized by the reduction in the number of chemotherapy courses with subsequent abatement of its related toxicity, both hematologic and nonhematologic, in a frail subset of patients.

## Figures and Tables

**Table 1:** Patient characteristics (*N*=55)

Median age, years (range)	70 (61–83)
*Sex*, N *(%)*	
Male	26 (47)
Female	29 (53)
*Symptoms*, N *(%)*	
A	39 (71)
B	16 (29)
*Bulky disease*, N *(%)*	
Yes	8 (14.5)
No	47 (85.5)
*LDH level*, N *(%)*	
Elevated	50 (91)
Normal	5 (9)
*Stage*, N *(%)*	
II	4 (7)
III	11(20)
IV	40 (73)
*aa-IPI score*, N *(%)*	
High intermediate (2)	30 (55)
High risk (3)	25 (45)

Abbreviations: aa-IPI, age-adjusted International Prognostic Index; LDH, lactate dehydrogenase.
